# Relating Amorphous Structure to the Tear Strength of Polylactic Acid Films

**DOI:** 10.3390/polym14101965

**Published:** 2022-05-11

**Authors:** Yutaka Kobayashi, Akira Ishigami, Hiroshi Ito

**Affiliations:** 1 Research Center for GREEN Materials and Advanced Processing (GMAP), 4-3-16 Jonan, Yonezawa, Yamagata 992-8510, Japan; akira.ishigami@yz.yamagata-u.ac.jp; 2 Graduate School of Organic Materials Science, Yamagata University, 4-3-16 Jonan, Yonezawa, Yamagata 992-8510, Japan

**Keywords:** biopolymers, tear strength, amorphous structure

## Abstract

Compared with polyolefins that are used as single-use plastics, polylactic acid (PLA) has a lower tear strength in films. The relationship between the tear strength and the higher-order structure of films was investigated using PLA films that absorbed moisture at 30 °C and 95% relative humidity (RH) or that had been annealed under reduced pressure conditions. Although the mobile amorphous (MAm) amount did not change under high humidity, the film became brittle due to enthalpy relaxation. The crystallization by annealing also caused embrittlement, and the MAm amount decreased to 10%. The displacement until tearing is lowered from 2.5 to 0.5 mm in both cases. However, in situ retardation measurements revealed that there was a significant difference in the fracture morphology of the torn tip. When crystallized, the molecular chains and crystals are oriented in the tensile direction of the film, and a fragmented structure is observed in the ligament. Embrittlement due to enthalpy relaxation caused a weak orientation perpendicular to the tensile direction of the film, and cracks occurs along with this orientation.

## 1. Introduction

Disposable packaging containers are often used for food distribution. In order to preserve food, packaging materials are required to be sterile and have gas permeability to prevent food spoilage. Petroleum-derived polymers such as polyolefins are inexpensive and are often used as disposable packaging materials. When used packaging is disposed of in the natural environment, these plastics take a long time to decompose into water and carbon dioxide. Additionally, when released into the ocean, it creates a great load on the ecosystem [1].

Polylactic acid (PLA) has been expected to replace polyolefins as a packing material because it is produced from biological raw materials [2]. PLA can be composted and decomposed easily in humid and warm conditions. However, even after one year in the ocean, PLA hardly shows any decomposition [3,4,5]. The marine environment has the following several characteristics that make it distinct from soil and compost: a low temperature, high salt content (conductivity), high pressure, and a low nutritional level (nitrate, etc.). In addition, there are fluctuations in different seasons or regions, and the water temperature also changes depending on the depth in the vertical direction. These characteristics also affect the activity of the microorganisms in the ocean.

On the other hand, commercial PLA consists of the following two enantiomers: L- and D-lactic acid, and the D-content is usually less than 5% for maintaining the crystallinity in a molded product. PLA needs to crystallize for thermal stability because food containers are frequently used in microwave ovens. When poly L-lactic acid (PLLA) and poly D-lactic acid (PDLA) are blended in equal amounts, stereocomplex (SC) crystals form [6,7,8]. A molded product with SC crystals has excellent heat resistance. However, the hydrolytic degradation of SC PLA is not faster than that of normal PLA, so utilizing PDLA does not solve the microplastic problem in the ocean at lower temperatures and with fewer microorganisms. When PLLA contains a few percent of D-form, the crystallization rate significantly reduces, and the hydrolysis rate increases. Generally, the presence of a trace amount of comonomer decreases the crystallinity of the random copolymer and changes its physical properties. In the case of high-density polyethylene (HDPE) and polypropylene (PP), their films are more flexible. According to the PE study [9,10], long-chain branches and high molecular weight components that prolong the relaxation time reduce the tear strength. Whether the crystallinity is the same in PE grades, PE with the smaller comonomer content exhibits a higher tear strength. As the D content in PLA increases, the tensile elongation increases, too. However, the elongation is much smaller than that of PE.

The reason for the brittleness of PLA is that the glass transition temperature (Tg) is approximately 60 °C, which is higher than room temperature. Even if L- and D-lactic acid are copolymerized or SC crystals form, Tg does not change. Thus, various strengthening methods have been studied for packaging containers. There are several methods that can be used to strengthen PLA [7,11,12,13], such as controlling the molecular structure of PLA by means of copolymerization with monomers other than lactic acid, blending plasticizers, or biodegradable polymers into PLA, and using natural materials as fillers [14]. Blending petroleum-derived polymers and fillers have even been attempted [15,16]. Furthermore, strengthening has been performed by utilizing polymer reactions such as cross-linking between different kinds of polymers, grafting reactions, and transesterification reactions. However, the degradability of this strengthened PLA in the ocean has scarcely been investigated.

Water is essential for the microbial and enzymatic degradation of PLA in the natural environment. During the process of hydrolysis, the higher-order structure of PLA is considered in a three-phase crystal, mobile amorphous (MAm), and rigid amorphous (RAm) model. The ratio of the three components changes during the process [17]. When hydrolyzed at 80 °C, which is a higher temperature than the Tg of PLA, it makes it difficult for the crystals to decompose, and the RAm structure promotes decomposition [18]. In our previous study, PLA films crystallized in the presence of water, even when the films that crystallized without water had the same crystallinity, and were susceptible to hydrolysis in a 70 °C and 95% RH environment [19]. Therefore, more detailed experiments are needed on higher-order structures and during hydrolysis.

As mentioned above, PLA has many problems, and we cannot say it is a marine-degradable polymer. However, in the development of food containers using marine-degradable polymers, which is our ultimate goal, we believe that it will be a starting point for finding a way to achieve both crystalline and degradable properties. Generally, a highly hydrophilic polymer is easily hydrolyzable. Therefore, increasing the toughness by absorbing water is the most appropriate method for improving the tearability of the packaging material. This study deals with the relationship between the higher-order structure of the PLA film, which is adjusted by water absorption and annealing, and the tear strength. In this study, we focused on the tear strength of packaging films such as plastic bags as an example of single-use plastics. The behavior of amorphous PLA in a constant temperature and humidity environment was quantitatively analyzed by the change in tear strength, which rapidly becomes brittle due to enthalpy relaxation. The deformation occurring at the tear tip was observed in situ using the latest instrument with an optical array. It is clarified that the mechanism of crack generation differs between the cases where PLA has crystallized and the case where PLA is embrittled by enthalpy relaxation.

## 2. Experimental Setup

For the PLA, Total Corbion (Rayong, Thailand) L175 with a melt mass flow rate (MFR) of 3 g/10 min at 190 °C was used. A compression-molding machine manufactured by Imoto Seisakusho (Kyoto, Japan) was used to prepare the film samples. The PLA pellets were pressurized at 6 MPa for 1 min after heating and melting at 190 °C for 4 min under reduced pressure; then, they were cooled at 20 °C for 2 min with a pressure of 6 MPa. The film thickness was about 100 μm. To change the higher-order structure, the PLA films were annealed at a predetermined temperature for 2 h under reduced pressure conditions in an AS ONE (Osaka, Japan) AW-250N vacuum oven. The constant temperature and humidity treatments were carried out at 30 °C and 95% relative humidity (RH) for a predetermined amount of time with a Tokyo Rika Kikai (Tokyo, Japan) KCL-2000W.

**Figure 1:**
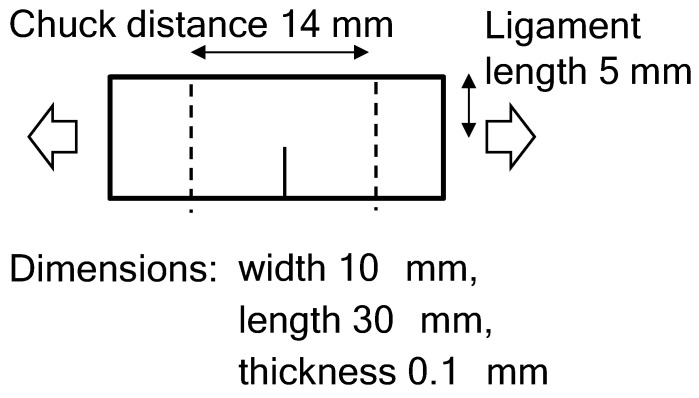
Illustration of the shape and dimensions of the film used for the tear test.

After adjusting the conditions of the film as stated above, a sample with the shape shown in Figure 1 was cut out from the film and used. A tear test was performed using a Sanko (Nagoya, Japan) ISL-T300 at a span of 14 mm and a tensile speed of 1 mm/min at room temperature. The measurement was repeated three times, and the average was taken as the reported value. A Photonic Lattice Inc. (Miyagi, Japan) WPA-micro and WPA-200 were used for the in-situ retardation measurements during the tear test to capture two-dimensional (2-D) mapping images of the retardation and the polarizing angles. The behavior of the tearing films was recorded by a video camera and then captured in a freeze-frame picture. After the tear test, the appearance of the torn films was taken using a microphotograph with a KEYENCE (Osaka, Japan) VHX-950F.

Thermal characterization was performed using differential scanning calorimetry (DSC) with a TA Instruments (New Castle, DE, USA) Q200. The same samples were used during the tear test. The change in specific heat (ΔCp) at the Tg was measured by increasing the temperature from 0 to 210 ° C at 10 ° C/min. The mobile amorphous (MAm) portion was quantified from the ratio of the change in the ΔCp at the Tg to the completely amorphous ΔCp 0.531 J/gK [20].

## 3. Results and Discussion

### 3.1. The Tear Strength of PLA after Conditioning in a Humid Chamber or an Oven

Polyolefins are non-polar and non-hygroscopic, and disposable plastic bags made from this material have a strong tear strength. On the other hand, PLA has high polarity and is easily affected by water. Thus, the film was conditioned at a high humidity of 95% RH and at 30 °C, and the changes in the tear strength were then measured.

**Figure 2:**
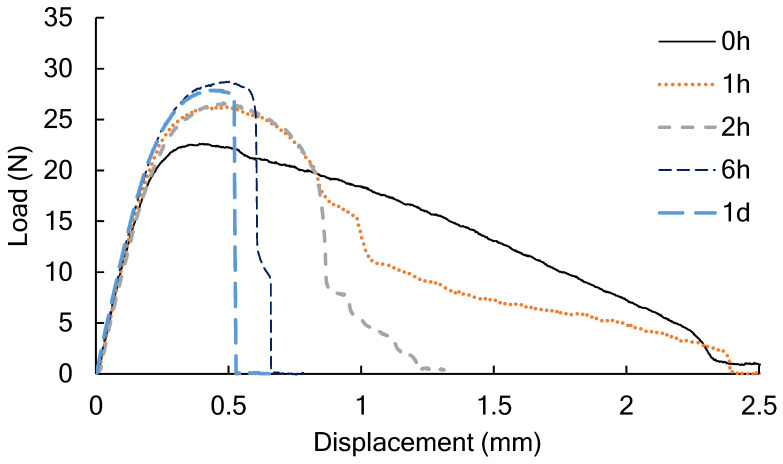
The load and the displacement of the crosshead of the PLA film conditioned in the humidity chamber for a predetermined amount of time from 0 h to 1 d in the tear test.

Figure 2 shows the load and the displacement of the crosshead during the tear test of the PLA film conditioned in the humidity chamber. Since the film thickness was unified to 100 μm, the maximum load indicated the difference in yield strength between films. Compared to the film (0 h) immediately after molding, the film that had been adjusted under high humidity conditions for one hour showed increased maximum strength, but the amount of displacement until fracture did not change. After conditioning for 6 h or more, the film was torn immediately after reaching the maximum load.

**Figure 3:**
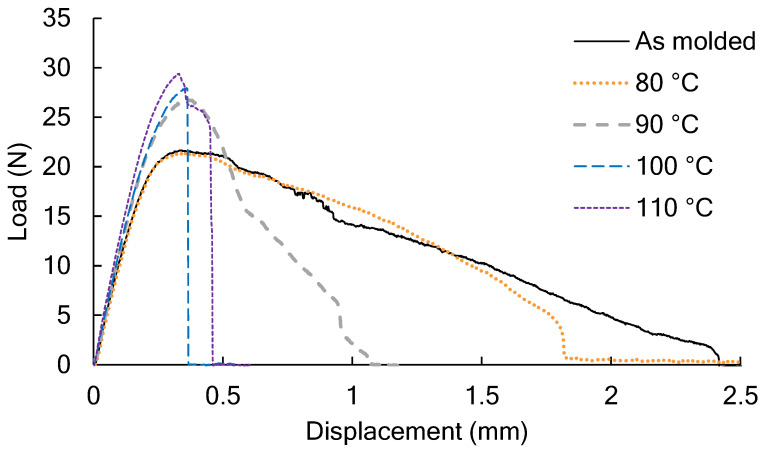
The load and the displacement of the PLA film annealed at a predetermined temperature from 80 to 110 °C under reduced pressure conditions in the tear test.

Figure 3 shows the load and the displacement in the tear test of the PLA film that was annealed at a predetermined temperature under reduced pressure. The reference sample (as molded) was prepared under the same conditions as the sample marked 0 h in Figure 1. After annealing at 80 °C for 2 h, the maximum load did not change, but the amount of displacement until tearing occurred decreased. When the annealing temperature was 90 °C or higher, the maximum load increased, and the amount of displacement decreased, and the tearability of the PLA film deteriorated.

### 3.2. The Appearance of Films after the Tear Test

The produced amorphous PLA film was transparent, and no changes were observed in the transparency, even when moisture below the Tg was observed. On the other hand, when annealing was performed at the Tg or higher, the transparent film became opaque as the annealing temperature increased. Despite these differences in appearance, the elongation at the ligament was uniformly reduced in the tear test. Thus, the torn film was observed under a microscope to determine the differences in the fracture morphology.

**Figure 4:**
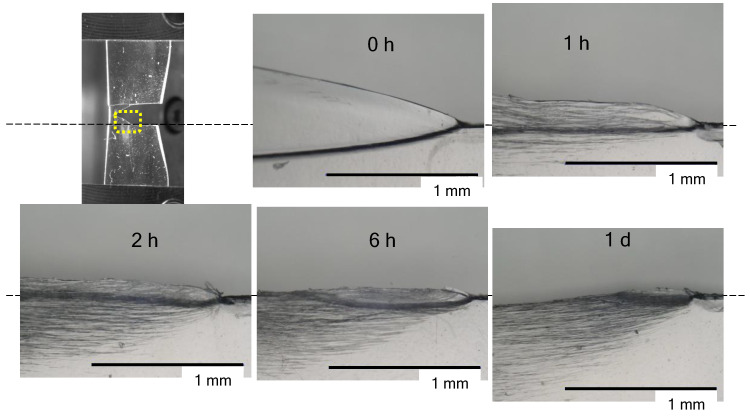
Photographs showing indications that there are tears in the film conditioned in a humidity chamber at 30 °C and 95% RH for a predetermined amount of time from 0 h to 1 d.

Figure 4 shows traces of tearing in the film that was conditioned in the humidity chamber at 30 °C and at 95% RH. The ligament of the 0 h film was necked and stretched into a thin film, and it was then cut off. After conditioning in the humidity chamber at 30 °C and 95% RH for 1 h or more, black flaws occurred parallel to the tearing direction; that is, tearing occurred in the direction perpendicular to the tensile direction. This black flaw is considered to be a large crack. As the conditioning time increased, the thin-film portion formed by necking became smaller, and the 1 d film had a brittle fracture morphology.

**Figure 5:**
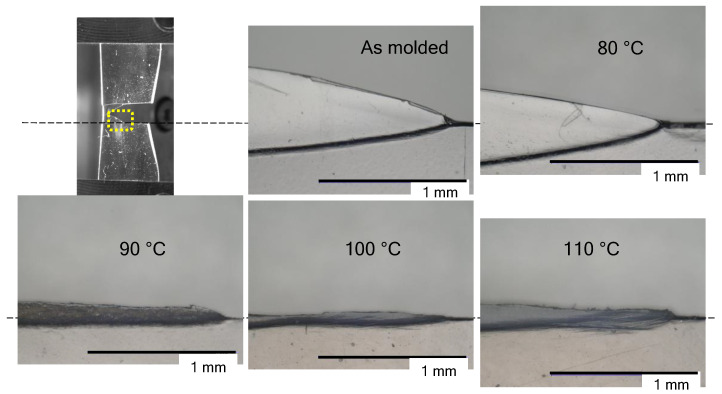
Photographs showing indications that there are tears in the film annealed at a predetermined temperature from 80 to 110 °C under reduced pressure.

Figure 5 shows traces of tearing in the film annealed under reduced pressure conditions. After annealing at 80 °C for 2 h, no changes were observed in the traces of tearing compared to the mold film. Necking and stretching were observed in the thin film. When annealing was performed at temperatures of 90 °C or higher, no necking deformation was observed. In addition, the black flaws that were originally observed in Figure 4 did not appear.

### 3.3. Morphological Changes at the Tip of the Tear

The fracture morphology of the torn film changed significantly depending on the pretreatment conditions of the film. Because PLA is a semi-crystalline polymer, crystallization is proceeded by annealing. Thus, it is natural for brittle fractures to appear after annealing. In the case of the water-absorbing PLA, no changes were observed in the transparency in spite of the embrittlement. As such, it can be inferred that the crack growth mechanism is different in the amorphous and crystalline states. Thus, using 2-D retardation mapping, we analyzed the distribution of the orientation and the direction of the fast axis of the birefringence ellipsoid [21,22].

**Figure 6:**
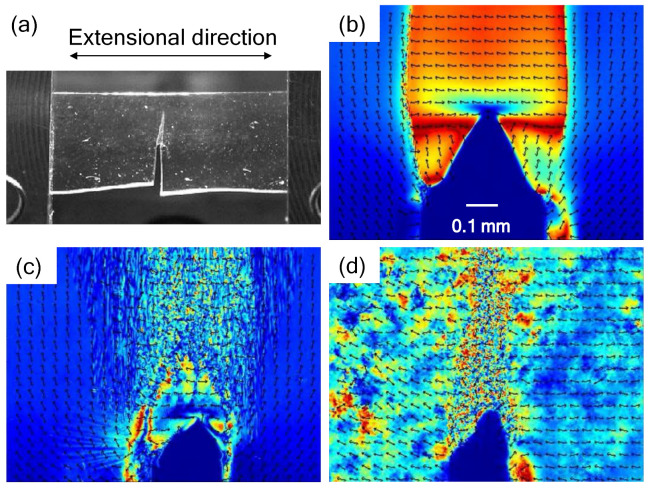
Images of the in-situ retardation measurements at the tip of the tear: (**a**) video capture, (**b**) 2-D mapping of the as-molded film, (**c**) 2-D mapping of the film-absorbed moisture after 6 h in a humidity chamber, (**d**) 2-D mapping of the film annealed at 100 °C for 2 h under reduced pressure conditions.

Figure 6 shows the distribution of the retardation at the tip of the tear and the direction of the high-speed axis of the birefringent ellipsoid, showing a displacement of approximately 0.4 mm. Figure 6a is a video capture image of the entire film. Necking occurred in the ligament, and it began to tear.

Figure 6b is the in-situ retardation mapping of the torn tip of the as-molded film during the tear test. The color change from ultramarine blue to dark red corresponds to the retardation changes taking place from 0 nm to 275 nm. When the film thickness is reduced by necking, the actual birefringence may be high due to the molecular orientation, but the observed retardation may be small due to a thinner thickness. Repeating retardation is also observed above 275 nm [22]. Although the film thickness was thin in the necking portion, the degree of retardation was large. That is, the stress was high during necking. The oriented direction coincided with the tensile direction. A local orientation perpendicular to the tensile direction was observed at the tip of the tear.

Figure 6c is the in-situ retardation mapping of the moisture that was absorbed by the film while in a humidity chamber for 6 h at 30 °C and at 95% RH. A striped pattern can be observed in the entire portion of the ligament that is perpendicular to the extension direction, and slight necking occurred at the tip of the tear. Furthermore, from the direction of the arrow indicating the high-speed axis, it can be seen that the oriented fragments are randomly spaced in the ligament.

Figure 6d shows the in-situ retardation mapping of the film that was annealed at 100 °C for 2 h under reduced pressure conditions. No uniform necking was observed in the ligament, and fragmentation occurred. As compared to (b) and (c), the orientation was parallel to the extensional direction at sites other than the ligament. From the comparison, it can be assumed that there is a relationship between the orientation perpendicular to the tension in (c) and the direction of the black flaws in Figure 4.

### 3.4. Changes in Heat Capacity at the Glass Transition Temperature

**Figure 7:**
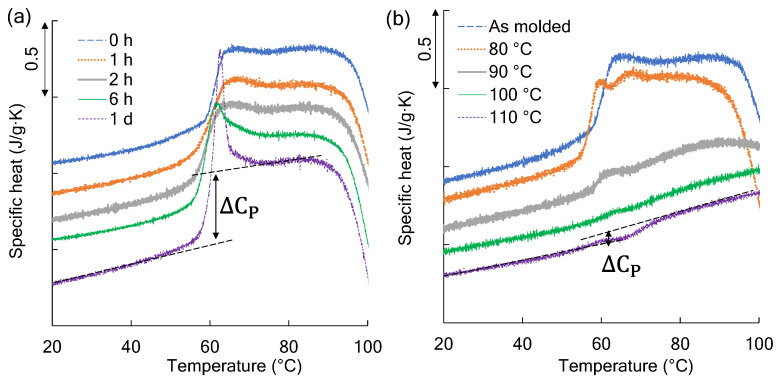
Changes in specific heat (ΔCp) near the glass transition temperature: (**a**) the film conditioned in a humidity chamber for a predetermined amount of time from 0 h to 1 d and (**b**) the film annealed at a predetermined temperature from 80 to 110 °C for 2 h under reduced pressure conditions.

Generally, the crystallinity increases and the amorphous portion decreases as the semi-crystalline polymer becomes more embrittled. Thus, using DSC, the amount of MAm was calculated from the change in ΔCp at the Tg. Figure 7a shows the change in ΔCp near the Tg of the film-absorbed moisture at 30 °C and at 95% RH for a predetermined time. As shown in Figure 4, a brittle fracture was observed on the PLA film, but the ΔCp hardly changed, indicating that the amount of Mam was constant. On the other hand, as shown in Figure 7b, when annealing was performed under the reduced pressure conditions, the ΔCp decreased, indicating that the amount of MAm decreased. Thus, in the tear test, the higher-order structure of the film was different, even in the films showing the same brittle fracture.

### 3.5. Effect of Water Absorption on the Tear Test

**Figure 8:**
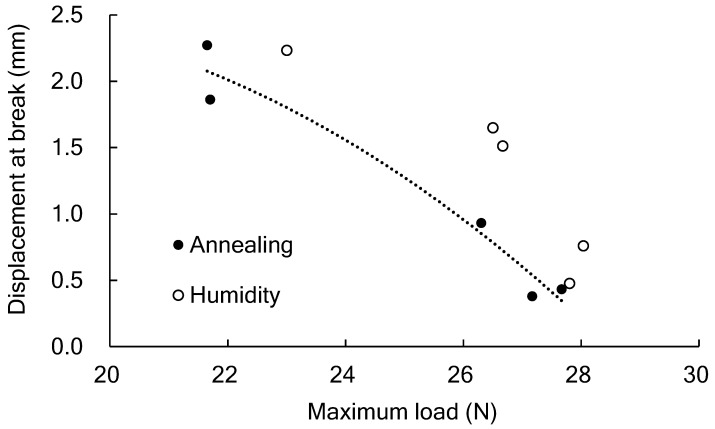
The relationship between the displacement at the breaking point and the maximum load measured in the tear test.

When PLA is used in plastic bags, there is a problem in that the tear strength is lower than that of existing polyolefins. Therefore, it is necessary to understand the tear behavior of PLA film. As shown in Figure 2 and Figure 3, the relationship between the displacement at the breaking point and the maximum load measured in the tear test is plotted in Figure 8. The as-molded film had a maximum load from 22 to 23 N and a displacement of 2 mm or more. When annealing was performed at the Tg temperature or higher under reduced pressure conditions, the maximum load increased to approximately 27 N, and the displacement decreased to approximately 0.5 mm. Via this annealing, the film changed from an amorphous state to a semi-crystalline state, with a crystallinity of 30% [19]. This increase in rigidity and the decrease in elongation associated with such crystallization are common.

On the other hand, the maximum load and the displacement in the tear test changed in the same way as they did during crystallization by annealing when the PLA film was conditioned in a humidity chamber at 30 °C and 95% RH. Engineered general-purpose plastics such as polyamide 6 and polyethylene terephthalate (PET) lose their rigidity as they absorb more water because water molecules act as plasticizers [23,24]. However, PLA becomes more brittle under high humidity conditions, as shown in Figure 4. The reason for this is the enthalpy relaxation that is indicated in the ΔCp at the Tg shown in Figure 7a. Although PLA is embrittled by enthalpy relaxation [25,26], the effects of water on enthalpy relaxation are unclear. From this study, it was clarified that PLA embrittles within one hour under high humidity conditions. Polycarbonate (PC) is an example when considering the cause of the rapid progress of PLA enthalpy relaxation due to water. PC has high toughness, but the ductile-brittle transition occurs due to enthalpy relaxation. When the temperature of physical aging closely approaches Tg, the yield stress increases in a short time, and then the brittle fracture occurs [25]. Even if the temperature of physical aging is Tg or less, the molecular chains move at a higher temperature, and PC reaches the thermodynamically stable state in a short time. It is considered that the physical aging of PLA is promoted by increasing molecular motion due to water absorption even at a constant temperature.

### 3.6. Influence of Mobile Amorphous on the Fracture Generation

**Figure 9:**
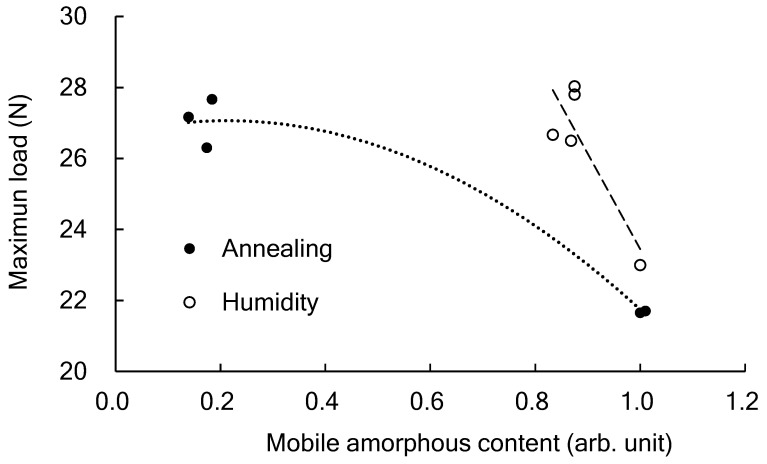
The relationship between the mobile amorphous amount (MAm) in the PLA film and the maximum load in the tear test.

Figure 9 shows the relationship between the amount of MAm in the PLA film and the maximum load in the tear test. The enthalpy-relaxed film has a higher amount of MAm than the film that was crystallized by annealing. Regardless of the differences in the amount of MAm, the film broke due to brittleness when the maximum load was about 27 N. Therefore, considering the practical use of PLA in plastic bags, no differences can be observed in the tearing characteristics regardless of the higher-order structure of PLA in the amorphous or semi-crystalline state. However, the behavior of fracture at the tip of the tear is very different between the two. In Figure 6d, the extensional direction and the orientation of the film coincide with each other. Generally, molecular chains or lamella crystals are arranged in the extensional direction. However, Figure 6c shows how the orientation is perpendicular to the extensional direction. In this case, the retardation that was generated in the film was very low, so the orientation of the amorphous molecular chains was small. Since this orientation coincides with the direction of the flaws and cracks generated around the ligament, it may indicate a pre-fracture stage such as the widening of the spaces between amorphous molecular chains.

## 4. Conclusions

The tear test was carried out at room temperature, which is lower than the Tg of PLA, meaning that the elongation in the break is smaller than that of polyolefins, which has a Tg that is lower than the test temperature. However, when using PLA as a packaging material, the essential problem is that the film ligament demonstrates little necking activity due to the embrittlement caused by crystallization or enthalpy relaxation. Therefore, in order to strengthen PLA, a polymer blend using a plasticizer that modifies the amorphous part and a biodegradable polymer with a low Tg that will strengthen the semi-crystalline part has been studied [7,11,12,13]. For polystyrene and ABS resin, which are synthetic polymers, techniques for modifying them using elastomers have been accumulated. However, a biodegradable elastomer with a high-impact modification performance has yet to be found. From this study, copolymers or a miscible polymer blend that slow down the enthalpy relaxation of PLA are also recommended as toughening techniques.

## Data Availability

The data presented in this study are available on request from the corresponding author.
